# Application of Metal–Organic Frameworks (MOFs) in Environmental Biosystems

**DOI:** 10.3390/ijms24032145

**Published:** 2023-01-21

**Authors:** Lu Zhang, Qingwen Zheng, Zheng Zhang, Huidong Li, Xue Liu, Jinzhi Sun, Ruiwen Wang

**Affiliations:** 1 Key Laboratory of Bio-Based Material Science & Technology, Ministry of Education, Material Science and Engineering College, Northeast Forestry University, Harbin 150001, China; 2 Key Laboratory of Micro-Systems and Micro-Structures Manufacturing, School of Life Science and Technology, Ministry of Education, Harbin Institute of Technology, Harbin 150001, China

**Keywords:** metal–organic frameworks, microbial electron transfer, biomass-derived material decoration, bioelectrocatalysis, biofuel cells

## Abstract

Metal–organic frameworks (MOFs) are crystalline materials that are formed by self-assembling organic linkers and metal ions with large specific areas and pore volumes. Their chemical tunability, structural diversity, and tailor-ability make them adaptive to decorate many substrate materials, such as biomass-derived carbon materials, and competitive in many environmental biosystems, such as biofuel cells, bioelectrocatalysts, microbial metal reduction, and fermentation systems. In this review, we surmised the recent progress of MOFs and MOF-derived materials and their applications in environmental biosystems. The behavior of MOFs and MOF-derived materials in different environmental biosystems and their influences on performance are described. The inherent mechanisms will guide the rational design of MOF-related materials and lead to a better understanding of their interaction with biocomponents.

## 1. Introduction

Metal–organic frameworks (MOFs) are porous, crystalline materials that are formed by self-assembling organic linkers and metal ions. Generally, the synthesis method of a MOF is determined by the type of metal or organic linker. Synthesis from the same reaction-starting materials may lead to MOFs with different structures and properties, and the synthesis methods and conditions will also affect their morphology, crystal structure, and porosity, thereby further affecting the function of the material. At present, there are many methods to prepare MOF materials with designable structures and excellent performance, such as hydrothermal/solvothermal synthesis, the ultrasonic method, the microwave heating method, the electrochemical method, and the mechanochemical synthesis method. All kinds of methods have advantages, which broaden the development and application of MOFs to a certain extent. The large surface areas and pore volumes of MOFs and their chemical tunability make them attractive for gas adsorption and separation [1,2], energy storage [3], catalysis [4,5], and sensing [6]. For example, Omar K. Farha and Taner Yildirim designed a new MOF material named NU-1501-M (M=Al or Fe). Because of its high specific surface area of 7310 m^2^/g, NU-1501-Al has a mass adsorption capacity of 0.6 g/g for methane gas at 10 MPa and 270 K. This material develops a better gas storage method for clean-energy vehicles [7]. In addition, due to different organic linkers and metal ions, MOFs can be divided into IRMOF (isoreticular metal–organic framework) series, MIL (materials of Institut Lavoisier) series, UiO (University of Oslo) series, PCN (porous coordination network) series, and ZIF (zeolitic imidazolate framework) series, etc. These can work alone or be integrated with others as a composite because MOFs usually are not three-dimensional, self-sustaining materials. While in most environment biosystems, large volume and block materials are typically needed for the large-scale of the related biosystems. Thus, the materials needed in these systems also should be large in volume and self-sustaining, and what is more, the proper structure should be supplied for microorganisms to grow on and ions to transport efficiently [8,9,10,11]. By compounding with other materials, all these requirements would be met. The substrate or structural material should be three-dimensional block materials with proper inner channels from several nanometers to several micron meters, biostable (long-term stability in biosystems), biocompatible, highly conductive (especially for bioelectrochemical systems), and easy to be decorated. As function components, MOFs can be synthesized in situ on the structural material or physically attached to it. Usually, the materials obtained by the in situ synthetic route are more uniformly distributed and more easily to contact with the reactants or the biocomponents compared to the physical mixture of different materials. Biomass-derived carbon materials are competitive as a substrate for these composite materials, as they process an inherent porous structure with multi-elements doping, high stability, good biocompatibility, and many kinds of functional groups as the active site [12,13,14], and can be easily modified by other materials, such as MOFs.

In natural environmental systems, chemical and biological reactions occur spontaneously and are responsible for elemental circulation and energy flow [15]. To better utilize these reactions, some artificial systems are also built for human benefit, such as wastewater treatment systems, biomass fermentation systems, bioelectrochemical systems, and biofuel systems (microbial fuel cells and enzyme fuel cells). Different efforts have been performed to promote the performance of these systems, such as building efficient engineering microorganisms, optimizing system design and operation parameters, and rational selective related materials [16]. Above all these approaches, materials are especially essential because they influence the hydraulic condition, microorganisms’ metabolisms, ionic transport, etc. We could construct substrate materials for the microorganisms to accommodate the inclusion site of the enzyme or for reactions to occur [17]. In addition, nanomaterials can be added as a functional additive to further improve the performance of these systems [18,19]. When MOFs are used as precursors, MOFs can be transformed into metal-based porous materials that are more stable than precursor MOFs and largely inherit the characteristics of precursor MOFs with a high specific surface area, structural diversity, and rich porosity. Due to the diverse composition and tunable structures of MOFs, they are considered to be suitable precursors for the preparation of porous materials. The usual preparation method is pyrolysis or solution infiltration under a certain atmosphere (for example, Ar, N_2_, air, etc.). Through the rational design of MOF precursors and the control of the synthesis process (such as gas atmosphere, pyrolysis temperature and time, heating rate and precursor addition, etc.), diversified MOF-derived materials that partially inherit the pore size, morphology, composition, and properties of MOF precursors can be prepared. Materials that are porous and chemically tunable are especially suitable for this proposal, such as MOFs and MOF-derived materials.

In this review, we will talk about the function and performance of MOFs and MOF-derived materials in environmental biosystems. The bioelectrocatalysis systems taken into account include enzymatic biofuel cells, microbial fuel cells, and also some fermentation systems. The interaction between these biocomponents and MOFs (or MOF-derived materials) is discussed, which will guide the rational design of MOF-related materials in environmental biosystems.

## 2. Environmental Bioelectrocatalysis Systems

The contents of environmental bioelectrocatalysis systems incorporate all electro-chemical processes that involved organism-catalyzed reactions. In this part, we mainly focus on bio-degradable macromolecule decomposition processes converting small and high-value substance or energy, but not the reverse ones, i.e., bioelectrocatalytic synthesis reactions. Bioelectrocatalysis can be defined as a series of phenomena that accelerate electrochemical reactions in the presence of biocatalysts, that is, using materials derived from biological systems as catalysts to catalyze redox reactions that take place at electrodes. Bioelectrocatalysis is an interdisciplinary study of electrocatalysis and biocatalysis, which fully combines the advantages of good activity, good selectivity, mild reaction environment of biocatalysis, and high energy conversion efficiency of electrocatalysis [20]. It is a technology that can be used to make high-value chemicals, clean biofuels, and new biodegradable materials in an environmentally friendly, sustainable, and efficient manner [21].

In this section, we focus on MOFs working in biofuel cells and evaluate their performance and inherent mechanisms. Biofuel cells mainly include enzymatic biofuel cells and microbial fuel cells, and these technologies are gaining attention for bioelectrocatalysis systems, which work by using oxidoreductases or electroactive microbial cells to catalyze the conversion of chemical energy into electrical energy [22]. Enzymatic biofuel cells offer high performance in low-power applications, such as portable medical devices, while microbial fuel cells excel in large-scale applications, such as energy-generating wastewater treatment. Actually, biofuel cells consist of many categories, and with changes in working biocomponents and configurations, the need for bioelectrocatalysis materials will change along with it. Still, there are some common needs for environmental bioelectrocatalysis systems, such as good biocompatibility, excellent electrochemical responses, high specific area, and proper pore structures. MOFs have been gaining much attention in bioelectrocatalysis due to their protective properties against enzymes and their large number of catalytic sites [23].

### 2.1. Enzymatic Biofuel Cells

The enzymatic biofuel cell (EBFC) is an energy conversion component that uses enzymes as catalysts and uses renewable biomass, such as glucose, ethanol, and hydrogen, as fuel to transform chemical energy into electric power [24]. During the whole process, the anodic substrate undergoes an oxidation reaction under the catalysis of the biologically active site [25], the lost electron has been transported to the cathode region via the external circuit, and the cathode substrate undergoes a reduction reaction under the catalysis of the biological active unit [26,27]. However, current EBFC performance still has shortcomings, such as a short life and low power density, which are difficult to meet the requirements of practical engineering applications. This is mainly because the amount of enzyme loaded onto the surface of the electrode is insufficient, which affects the electrocatalytic efficiency and current density of the enzyme electrode; the enzyme generally has a nonconductive protein shell, which hinders the electron transfer between the enzyme catalytic center and the electrode surface. The enzyme is easy to fall off and inactivate, which makes the stability of the enzyme electrode poor [28]. Therefore, it is necessary to design an enzyme electrode with high electron transfer efficiency, low resistance of the modified layer, high enzyme loading capacity, firm enzyme fixation, and high activity. The high specific surface area, extremely high porosity, and tunable pore size of MOFs can improve enzyme loading [5]. Their various functional groups can increase the adsorption capacity of MOFs to firmly adsorb immobilized biomolecules or electroactive molecules. In addition, their high chemical stability ensures its structural integrity under different conditions.

Li et al. have successfully prepared highly flexible BC/c-MWCNTs/ZIF-8@LAC electrodes by encapsulating laccase (LAC) in zeolitic imidazolate framework-8 (ZIF-8) and combining it with bacterial cellulose (BC)/carboxylated multiwalled carbon nanotube (c-MWCNT) and applied the electrode in a single-enzymatic biofuel cell (EBFC). Despite five cycles of use, the designed device shows excellent sustainability with high operating voltage and power density. Encapsulation of the enzyme within ZIF-8 provides excellent protection of the enzyme under a variety of conditions. In addition, the single-enzyme biofuel cells (EBFCs) demonstrated a rigid linear correlation from 0.01 to 0.4 mM, and the detection threshold was as low as 1.95 × 10^−3^ mM for bisphenol A [29].

**Figure 1:**
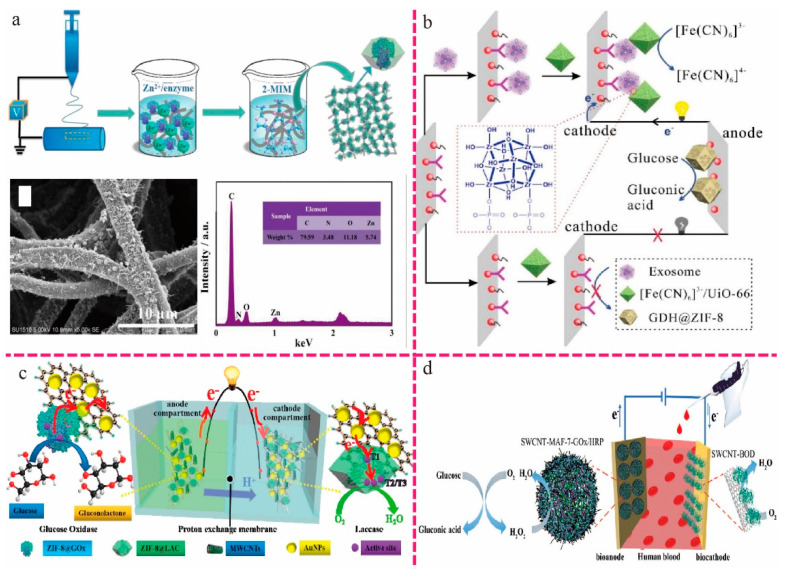
(**a**) Schematic diagram of PU/RC/ZIF-8@enzyme synthesis [30]. Copyright 2022, Elsevier. (**b**) Illustration of the principle of self-powered BFC-based biosensors in the presence or absence of exosomes [31]. Copyright 2021, Elsevier. (**c**) Schematic of self-powered glucose biosensor based on EBFCs [32]. Copyright 2021, Elsevier (**d**) Schematic of the fabrication of an SWCNT and GOx/HRP coembedded MAF-7 biocatalyst [34]. Copyright 2020, American Chemical Society.

Wei et al. synthesized polyurethane/regenerated cellulose (PU/RC) nanofibers by electrospinning in situ-grown GOx-encapsulated ZIF-8 particles and then mixing them with CNTs to prepare PU/RC/ZIF-8@GOx/CNTs electrodes, as shown in Figure 1a. Immobilized enzymes are the key components of wearable EBFCs; therefore, improving the efficiency of immobilized enzymes is an effective way to improve energy efficiency. They used the ZIF-8 enzyme encapsulation strategy to improve the immobilization, environmental stability, storage, and reusability of enzymes. The maximum power density output of the designed EBFC in the natural state is 1.09 W/m^3^ when the elongation is less than 15%; the voltage is relatively stable without a significant drop. However, if the elongation continued to increase to 25% and 50%, larger voltage drops were observed but still remained at a high level of 88% compared to the initial voltage. Under stretching, bending, and twisting several times, the stretchable enzymatic biofuel cell (EBFC) produced a stable energy output, indicating that the designed device could serve as a reliable energy supply for the use of wearable systems [30]. Gu et al. used bimetallic organic frameworks as carriers for biological enzymes and electroactive probes. The GDH anodic enzyme immobilized in ZIF-8 displayed better catalytic activity and temperature and pH tolerance, making the biosensor more stable. Moreover, a zirconium metal–organic framework (UiO-66-NH_2_) with electroactive molecules (K_3_[Fe (CN)_6_]) acted as a nanoenrichment carrier, improving the efficiency of the cathode, which further increased the sensitivity of the biosensor. This resulted in a detection limit of 300 particles/mL (3 s/k) for the as-prepared biosensor (Figure 1b) [31]. Li et al. encapsulated an enzyme in ZIF-8 and the situ grew ZIF-8 on an electrospun CA nanofibrous membrane. The CA/ZIF-8@enzyme/MWCNTs/Au membrane was used as self-energizing glucose highly flexible electrode for the biosensor. At the same time, the glucose biosensor had good long-term stability in continuous operation for up to 15 h (Figure 1c) [32]. 

Liang et al. successfully synthesized novel porous hollow tubular carbon materials (PHTCs) by one-step pyrolysis of zeolite imidazolate framework-8 derived from ZnO nanorods (NRs) with ZnO on its surface. Nucleation and growth sites were generated, and ZIF-8 was grown by providing 2-methylimidazolium salts as ligands. To improve the electron conductivity, PHTC was further modified with Au nanoparticles (NPs) to obtain PHTC@Au. Finally, the enzyme anode was successfully constructed using PHTCs@Au as the carrier material. The as-prepared BFC assembled with the PHTCs@Au/GOD-GA/GC electrode as the bioanode and the commercial platinum plate electrode as the cathode obtained a maximum power density of 310 μW/cm^2^ at 0.23 V, and the open circuit potential was 0.63 V [33]. Zhu et al. constructed a single-walled carbon nanotube (SWCNT) and cascaded enzyme-glucose oxidase (GOx)/horseradish peroxidase (HRP) coembedded hydrophilic MAF-7 biocatalyst (SWCNT-MAF-7-GOx/HRP). Benefiting from the effective protection of the enzyme by MAF-7, the SWCNT-MAF-7-GOx/HRP electrocatalyst is much more resistant to temperature and small organic molecule inhibitors than the unprotected enzyme. Furthermore, the electrocatalytic activity of hydrophilic SWCNT-MAF-7-GOx/HRP exceeds that of SWCNT-ZIF-8. In human whole blood, the SWCNT-MAF-7-GOx/HRP catalytic EBFC showed an eight times higher power density and 13-fold higher stability compared to unprotected enzyme-based EBFCs (Figure 1d) [34]. 

Although efforts have been done in over the years, there are some aspects that need to be addressed in future research. In terms of mechanism research, the calculation at the atom level should be taken into consideration, which would help to predict the performance of unreported MOFs in EBFC. From the future perspective of commercialization, the cost of synthesized MOFs should be further cut down.

### 2.2. Microbial Fuel Cells

At present, our society is experiencing several major challenges, such as climate change [35], water scarcity [36], and energy shortages [37]. Fuel cells are efficient and environmentally friendly conversion devices with a wide range of applications [38]. Microbial fuel cells (MFCs) are an attractive technology due to their technical viability and environmental friendliness [39]. In MFC reactors, electrochemically active microorganisms accommodate on the anode, convert biodegradable organic matter into CO_2_, and secrete electrons while electrons and protons are transferred to the cathode chamber to fulfill this cycle. For microbial fuel cells, MOFs can be used both at the anode and also the cathode. There are different needs for different electrodes.

Usually, what occurs in the cathode is an oxygen reduction reaction (ORR), which is ecofriendly and economically efficient [40]. ORR has been studied for many years; however, when it comes to the cathode of MFC, things are much more complicated, as the reaction occurs at a three-phase interface and microorganisms are involved. The oxygen in the air diffuses into the cathode surface followed by the catalytic reduction of oxygen and, at the same time, electron transfer from the cathode to the oxygen at the interface. MFCs are highly limited in their development and practical application due to the high overpotential and slow reaction kinetics of ORR [41]. Pt-based catalysts have been demonstrated as efficient ORR catalysts in MFCs, but their low stability and high price hinder them from being widely used in commercial applications. Among the air cathode MFCs, their low cost, simple construction, and naturally abundant source of oxygen have attracted extensive attention from research for commercial application. The development of noble-metal-free electrocatalysts that are stable and have outstanding ORR performance is highly desirable. Due to their well-developed inner structure, proper interfacial properties, large specific surfaces, and unsaturated metal ion active sites, MOFs have exhibited promising potential for electrocatalytic applications [42].

There are many works that describe the excellent performance of MOFs used as oxygen reduction catalysts [41] (Table 1), but most of these studies were conducted in alkaline solutions, and usually, there are no microorganisms in these systems, which are quite different compared with MFCs (pH neutral and with biocomponents). In addition, the biocomponents would bring out new challenges, such as catalyst passivation caused by complex ions or bio-macromolecules produced by microorganisms. Nevertheless, the application of MOFs as MFC electrode materials is still far from being fully understood because the vast quantities of MOFs consist of inactive organic ligands, and they are electrical insulating. It is currently possible to improve the electrical conductivity of MOFs by using carbonization strategies.

Priya Mukherjee et al. synthesized carbon nitrogen frameworks (Co-CNF-1 and Co-CNF-2) after high-temperature pyrolysis of Co-Zn ZIF with and without silica protection. According to the study, protecting the MOF surface with a silica coating prevents aggregation and provides uniform heating, resulting in improved reduction capability. Both CNFs showed high conductivity, surface area, and cobalt concentration, which benefited ORR, and according to the electrochemical study, both CNFs followed four e-transfer pathways that also were beneficial for ORR. In the MFC of the CNF cathode, the maximum power density was 1.1 times of the Pt/C cathode, and the COD removal in the case of the aerated tap water catholyte was also higher than the Pt/C cathode [52]. Bonyoung Koo et al. synthesized ZIF-67U and ZIF-67H cathodes by combining activated carbon and ZIF-67 through ultrasonication (U) and solution precipitation (H). There was an increase of 60% and 48% in maximum power density between the ZIF-67U cathode and ZIF-67H cathode compared to the AC cathode, and it was 160% and 140% higher than that of platinum cathode, respectively. This is due to the fact that the cobalt nitrogen of ZIF-67 U and ZIF-67 H enhanced oxygen ORR active sites, improved reaction rates, and decreased charge transfer impedance [53].

**Figure 2:**
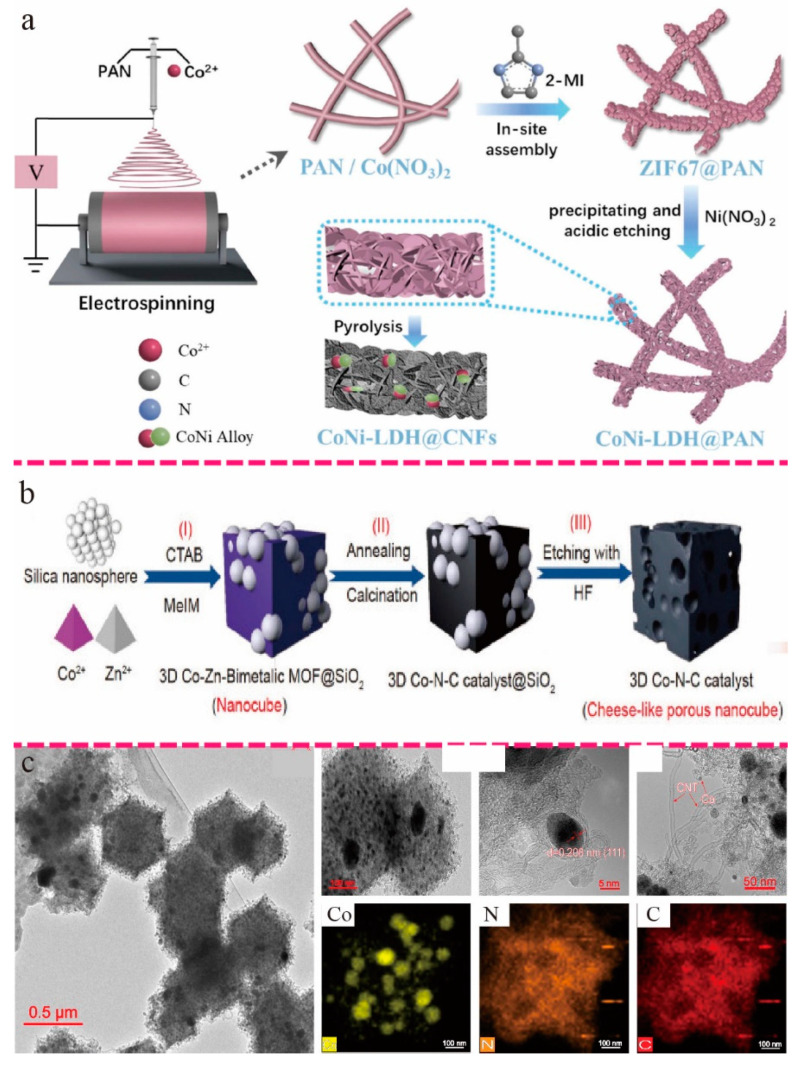
(**a**) Schematic illustration of CoNi-LDH@CNFs synthesis [47]. Copyright 2022, Elsevier. (**b**) Three-dimensional hierarchically porous Co-N-C skeleton synthesis diagram [54]. Copyright 2021, Springer. (**c**) FESEM images of ZnCo-ZIFs-derived Co-NCNTNP and elemental mappings of Co, N, and C in Co-NCNTNP [51]. Copyright 2019, Elsevier.

**Figure 3:**
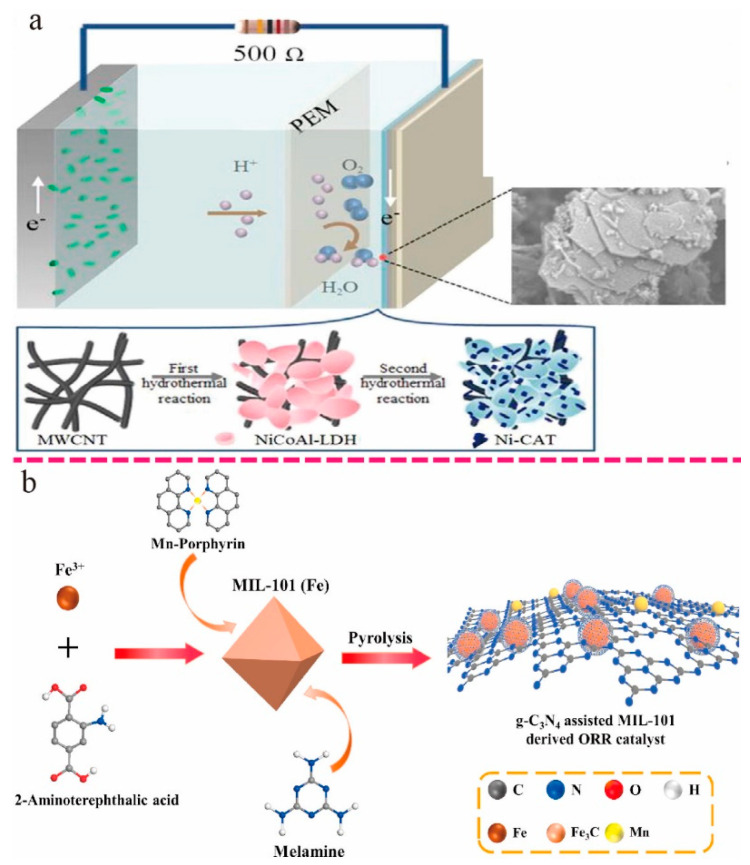
(**a**) Schematic of MFC and the preparation process of the Ni-CAT/NiCoAl-LDH/MWCNT composite [50]. Copyright 2021, Elsevier. (**b**) Schematic illustration of the fabrication of the Mn–Fe@g-C_3_N_4_ electrocatalyst [49]. Copyright 2020, Elsevier.

Li et al. fabricated nanoflower dendritic composites (CoNi-LDH@CNFs) with rich concave structures constructed from electrospun nanofibers. CoNi-LDH@CNFs exhibited excellent ORR catalytic activity in the company of the highest power density of 1.39 W/m^2^, which was greater than the corresponding Pt/C MFC (0.844 W/m^2^) (Figure 2a) [47]. Chen et al. reported a synthetic route in which a bimetal–organic framework (bi-MOF)-derived Swiss cheese-like carbon was obtained with a novel 3D hierarchical porous framework (3D Co-N-C). This was achieved with self-regulated thermal decomposition of Co/Zn bi-MOFs using cetyltrimethylammonium bromide (CTAB) as a structure-orienting surfactant that could control the morphology of the cubic framework. In addition, the silica spheres served as a template and contributed to the hierarchical porous structure. Compared with the commercial Pt/C-MFC anode (maximum power density of 1078 mW m^−2^), the 3D CoNC-MFC device exhibited better power performance (1257 mW m^−2^) and had excellent stability (Figure 2b) [54]. Zhang et al. synthesized a novel nitrogen-doped carbon nanotube-embedded cobalt nanoparticles nanopolyhedra (Co-NCNTNP) electrocatalyst by carbonizing bimetallic metal–organic frameworks. The obtained materials were integrated with the activated carbon and used as air-cathode microbial fuel cells (MFCs). The Co-NCNTNP-MFC device obtained a higher maximum power density (2252 mW/m^2^) than AC-MFC (888 mW/m^2^) and Pt/C-MFC (1260 mW/m^2^) (Figure 2c) [51]. Chen et al. designed a composite that combined NiCoAl-layered double hydroxide (LDH) nanosheets, Ni-catecholate-based metal–organic framework (Ni-CAT MOF), and multiwalled carbon nanotubes (MWCNTs). The maximum plateau voltage of Ni-CAT/NiCoAl-LDH/MWCNTs was 0.48V, the longest cycle duration was 8 days, and the maximum power density was 448.5 mW/m^2^, which is 1.03 times that of NiCoAl-LDH/MWCNTs-MFC (433.5 mW/m^2^), which is 1.35 times that of NiCoAl-LDH-MFC (329.9 mW/m^2^). The unique structure of LDH, the high electrical conductivity of Ni-CAT, and the MWCNTs enhanced the ion transportation between layers and significantly reduced the resistance, thereby effectively improving the electrode’s long-term stability and power output (Figure 3a) [50].

Zhong et al. proposed a new strategy for the preparation of hierarchical porous bimetallic carbon nanofibers (Mn–Fe@g-C_3_N_4_) by pyrolysis of Mn-doped g-C_3_N_4_-assisted Fe-based MOFs (MIL-101). As a class of iron-based nitrogen-rich MOF, MIL-101(Fe) has a well-organized pore system and abundant unsaturated metal ion active sites. Mn-Fe@gC_3_N_4_-MFC has a maximum power density of 413 mW/m^2^, which is 1.24 times that of MFC with a Pt/C cathode (Figure 3b). The excellent catalytic activity of Mn–Fe@g-C_3_N_4_ is mainly attributed to the 3D interconnected porous structure, highly conductive framework, and synergistic effects between nitrogen and metal ion centers [49]. Priya Mukherjee presented a PEDOT-modified MIL-53 (Al) electrocatalyst for improved cathode half-cell potential in MFCs. Electrochemical characteristics of the synthesized modified MOF showed a four-electron transfer pathway, which is beneficial to enhance the electrocatalytic efficiency of the cathodic ORR process. In addition, an MFC with P-MIL cathodes achieved a maximum power density of 4780 mW/m^3^ [55]. Bimetallic NH_2_-UiO-66(Zr/Ni) was synthesized using a hydrothermal process by Md Tabish Noori et al., and NH_2_-UiO-66(Zr/Ni) displayed a higher ORR activity than Pt-C. The incorporation of Ni as a secondary metal node in MOF significantly increased ORR peak current and reduced charge transfer compared with a single-metal node (Zr) MOF and 10% Pt-C cathode due to improved molecular alignment and surface porosity resistance. The MFC with NH_2_-UiO-66(Zr/Ni) cathode achieved a high-power density (800 mW/m^2^) and had excellent coulombic efficiency (75%) [56].

In these research papers, as MFCs cathode catalysts, MOFs had shown creditable and excellent performance and long-term stability. However, long-time behaviors of MOFs remain a topic worth further discussing. The status of MOFs and the respective microbial community structure should be described since it has been reported that some bacteria could also function as oxygen reduction catalysts. After long-time operation, it is not surprising that cathodes are covered by thick biofilms, which influences ion transport, oxygen diffusion, and also the physical and chemical properties of MOFs. Detailed distinction and description of the performance between these biofilms and MOFs will help us obtain a better knowledge of the behavior of MOFs over time.

There are also several reports about MOFs used as anode materials. Generally speaking, as anode materials, they should have a proper pore structure to colonize microorganisms and to absorb electron shuttles. What is more, the chemical composition of anode materials would also influence the performance of an MFC because many electrochemical active bacteria are metal-reduction bacteria that can use metal or metal ions as electron mediators. Thus, the metal component in MOFs also makes them competitive as an MFC anode, and it is likely that Fe and Mn are more favorable for these electrochemical active bacteria. These metals not only influence electron transfer but also shape the microbial community structure and, in return, influence the performances of MFCs.

Li et al. reported pyrolyzing Fe-MIL-88B-NH_2_ modified seitan composites, and hierarchical porous carbon foams (HPCFs) are formed, which can form 3D free-standing MFC anodes. A maximum power density of 11.2 W m^−3^ and a current density of 23.1 A m^−3^ were achieved, benefiting from the macroporous structure and good biocompatibility. As a consequence, bacterial adhesion and charge transfer efficiency between bacteria and electrodes would be enhanced. In addition, with abundant pore structures, MOFs could also adsorb electron mediators secreted by exoelectrogens, thus promoting electron transfer [57]. Hu et al. grew 2D Zn-Fe-MOF arrays on commercial carbon cloth and fabricated a nano-Fe_3_C@2D-NC@CC anode through carbonization in which nano-Fe_3_C particles vertically stood on N-doped carbon arrays. This anode was binder-free. Assembled into a mix-culture MFC, it exhibited a significant improvement in electrocatalytic activity in bacteria and provided MFCs, and it had a maximum power density of 1606 mW m^−2^ compared to 583.3 mW m^−2^ of a commercial carbon cloth anode. The mechanisms of MOFs facilitating electron transfer in an anode interface may result from the increase of both direct electron transfer and indirect electron transfer. The direct electron transfer may be enhanced by the interaction between MOFs and cytochromes, but this could only occur in the monolayer of microorganisms near the electrode, whereas other microorganisms far away would transfer electrons mainly through indirect electron transfer mediated by electron shuttles, which may be absorbed and facilitated by MOFs [58].

The current research mainly focuses on enhanced performance but little discussion has been completed regarding extracellular electron transport. Future research should dig into microbial behavior with the addition of MOFs, long-term changes of MOFs, and the succession of the microbial community.

## 3. Bioanaerobic Conversation Systems

Anaerobic conversion of organic compounds, especially from biomass or waste, is important in high-value chemical acquisition and biogas energy conversion. How to improve the biogas production and the efficiency of chemical content in bioanaerobic conversation systems is a current focus. Commonly, these systems are a microbial community of anaerobic bacteria and archaea. For example, in a methane production system such as an anerobic digestion (AD) system, anaerobic bacteria metabolize acid into hydrogen, and methanogenic archaea convert hydrogen into methane [59]. During this process, interspecies electron transfer (IET) occurs between these microorganisms. Electron or electron shuttles (CH_4_, quinone and fumaric acid, etc.) circulate between different species in IET [60,61]. Adding MOFs into these systems, on one hand, would intensify the interaction between different species. On the other hand, the production of high-value chemicals or biogas may be enhanced by improving the metabolisms of microorganisms [62]. 

**Figure 4:**
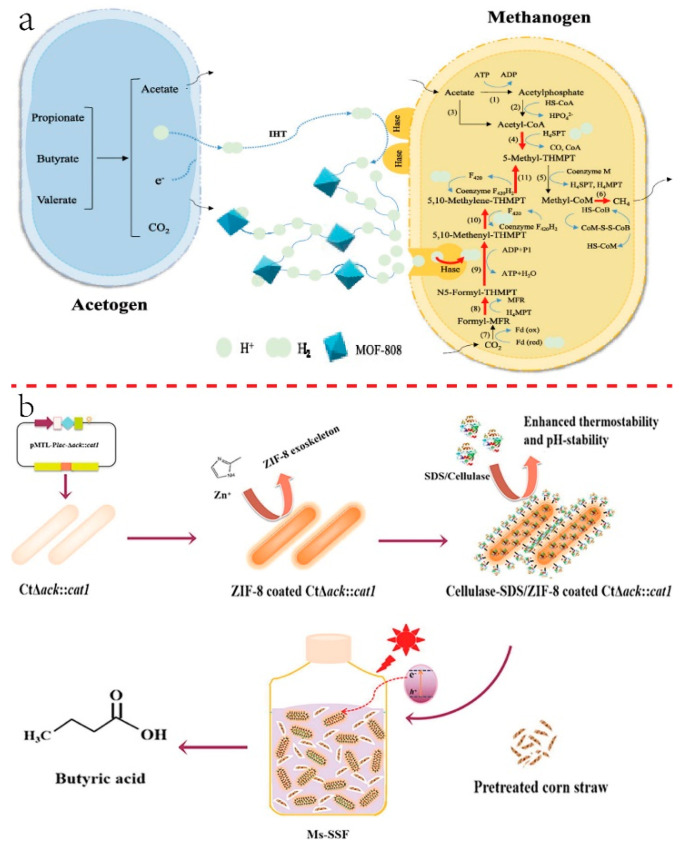
(**a**) Schematic illustration MOF-808 facilitating IET [63]. Copyright 2022, American Chemical Society. (**b**) MOFs served as a photocatalyst and protective exoskeleton for cellulase in the hydrolysis of rice straw and butyric fermentation system [66]. Copyright 2021, Elsevier.

Chen et al. added MOF-808 into the anaerobic digestion system of corn stalks, and the biogas production and methane content were increased from 11% to 40% and 10 to 14%. The addition of 500 mg/L MOF-808 was optimal for biogas production. The mechanisms are that the hydroxyl of MOF-808 can offer protons according to the protonation in this system, thus forming a hydrogen bond network assisting methane rapidly transport through the channel of the MOF-808. Moreover, it influenced the microbial community, hydrogen metabolism level of methanogens, and interspecies hydrogen transfer, which both contribute to promoting methane production (Figure 4a) [63]. Another research work reported that MOF-808 was used in this system and helped to convert waste-activated sludge (WAS) into CH_4_. By breaking through the limit through the poor hydrolysis of sludge and/or poor syntrophic methanogenesis in this system, the methane production increased by approximately 26.7% (the proportion of methane was 15.6%) with the addition of 150 mg MOF-808/g. Results also showed MOF-808 not only enhanced the enzymatic hydrolysis of sludge but also functioned during the abiotic hydrolysis of sludge extracellular organic substances. What is more, MOF-808 also enriched hydrogen-producing bacteria and methanogens (i.e., Methanosarcina) in the microbial community. Highly efficient syntrophic methanogenesis was achieved by altering the methanogenic pathway (promoting proton transfer between syntrophic species, especially accelerating the formation from carbon dioxide to methane) [64]. A zeolite imidazolate framework-67(ZIF-67)-derived porous carbon (PC) was used in anaerobic digestion, in which the highest biomethane yield (614.0 mL/g) from ethanol was obtained with 100 mg/L PC. PC provided a microbial electron transfer highway; thus, interspecies electron transfer was improved, and free charge transfer resistance was reduced. What is more, decreased expression of functional genes related to nanowires and cytochromes was observed. The addition of PC-800 also enhanced the secretion of redox-active humic substances (HSs) and tightly bound EPS (TB-EPS) [65].

Liu et al. established a system that coupled simultaneous rice straw hydrolysis and butyric acid production. MOFs (ZIF-8) were added and used as a photocatalyst and protector for cellulase during the hydrolysis of rice straw. Improvement of butyric acid production of 0.41 g/L h was observed in fed-batch fermentation as well as 55% of rice straw hydrolyzation in 24 h [66].

These reported fermentation systems are different in organics conversations and microbial communities. The behaviors of anaerobic bacteria and archaea influenced by MOFs are discussed; however, there is little information about the fungus in these systems, which would also contribute to the production of chemicals. What is more, besides the production of chemicals, standard methods should be established when evaluating different MOFs used in different anaerobic systems, and this will help the future design of efficient catalysts in other anaerobic systems.

## 4. Conclusions

Environmental biosystems are important in element circulation, energy production, chemical conversion, etc. Various approaches and materials are conducted to improve their performance and study their related inherent mechanisms. Large specific areas and active sites of materials can offer a sufficient site for biochemical reactions and microorganism accommodation. MOFs and related materials are porous and chemically tunable, thus making them competitive for these systems. They can catalyze central reactions (such as an ORR in MFC cathodes, an enzymatic reaction in EBFC, and so on), facilitate interspecies electron transfer (e.g., anerobic systems), and improve their performance. Their usage and functions are summarized in Table 2. Moreover, MOFs are also an ideal material to study the interaction of the interfacial behavior in these systems. Although several research papers have been completed on these systems, the interaction between MOFs and biocomponents is still not fully understood. Firstly, only several types of MOFs are investigated in bioelectrochemical or anaerobic digestion systems, and more investigation needs to be conducted. Secondly, research on MOFs interacting with a single bacterial cell is deprived, especially for those functional microorganisms in these systems, for example, electroactive bacteria or methane-producing archaea. To further understand MOFs functioned in these systems, a better understanding of local and real-time characterization and technology needs to be developed, and this platform will capture subtle differences in responses from biocomponents to MOFs. Many untapped opportunities still exist, including the development and application of MOFs and MOF-derived materials with large mesopores or redox-active or conjugated systems to enhance the conductivity and even selectivity of MOF layers. Such systems are expected to facilitate bioelectrocatalytic reactions, for example, by increasing electron transfer rates and/or mass transport. Further theoretical and experimental studies are needed to better understand the encapsulation of biocatalysts, the effect of pore size on mass transfer, and the stability, degradation, and cytotoxicity in aqueous environments to further comprehensively improve bioelectrochemical devices. These approaches and improvements will provide comprehensive insights for understanding the mechanisms among MOFs and the biocomponent in these systems and lead to a better design of related materials.

## Figures and Tables

**Table 1:** MOFs used as MFC cathode materials and their performances.

Cathode Catalyst	Anode Volume (mL)	Average Operating Voltage (mV)	Open-Circuit Voltage (mV)	External Resistance (Ω)	Maximum Power Density (mW/m^2^)	Ref#
Zr-MOF	150	305 ± 59	891	100	131.2 ± 3.5	[43]
MOF-800 (Cu-bipy-BTC)	43	365	588	500	326 ± 11	[44]
Ni/CoNC (Co-MOF)	100	300	600	1000	4335.6	[45]
BGQDs/MOF-15	28	600	680	1000	703.55	[46]
CoNiLDH@CNFs (ZIF-67)	-	410	641	1000	1390.37	[47]
Co3O4/NiCo2O4 (ZIF-67)	28	-	252	1000	1810	[48]
Mn–Fe@g-C3N4 (MIL-101)	350	450	568	1000	413 ± 7	[49]
NiCAT/NiCoAl-LDH/MWCNT (Ni-CAT MOF)	-	475	667	-	448 ± 12	[50]
Co-NCNTNP (ZnCo-ZIFs)	28	-	297	1000	2252 ± 46	[51]

**Table 2:** MOFs usage and their functions.

Type of MOFs	Environmental System	Function	Ref#
BC/c-MWCNTs/ZIF-8@LAC	EBFC	protection	[29]
PU/RC/ZIF-8@GOx/CNTs	EBFC	Immobilized enzymes	[30]
UiO-66-NH2	Biosensor	carriers	[31]
CA/ZIF-8 @enzyme/MWCNTs/Au	Glucose biosensor	protection	[32]
PHTCs@Au/GOD-GA/GC (ZIF-8)	BFC	protection	[33]
SWCNT-MAF-7-GOx/HRP	EBFC	protection	[34]
Zr-MOF	MFC cathode	catalyst	[43]
MOF-800 (Cu-bipy-BTC)	MFC cathode	catalyst	[44]
Ni/CoNC (Co-MOF)	MFC cathode	catalyst	[45]
BGQDs/MOF-15	MFC cathode	catalyst	[46]
Co3O4/NiCo2O4 (ZIF-67)	MFC cathode	catalyst	[48]
Mn–Fe@g-C3N4 (MIL-101)	MFC cathode	catalyst	[49]
NiCAT/NiCoAl-LDH/MWCNT (Ni-CAT MOF)	MFC cathode	catalyst	[50]
Co-NCNTNP (ZnCo-ZIFs)	MFC cathode	catalyst	[51]
Fe-MIL-88B-NH2	MFC anode	promoting electrons transfer	[57]
Fe3C@2D-NC@CC (Zn-Fe-MOF)	MFC anode	promoting electrons transfer	[58]
MOF-808	Anaerobic digestion	offer protons	[63]
MOF-808	Enzymatic hydrolysis of sludge	enriched hydrogen-producing bacteria	[64]
ZIF-67	Anaerobic digestion	improved interspecies electron transfer	[65]
ZIF-8	Rice straw hydrolysis and butyric acid production	photocatalyst	[66]

## Data Availability

Not applicable.
